# Supplementary material for: Addition of Chromosome 17 Polysomy and HER2 Amplification Status Improves the Accuracy of Clinicopathological Factor-Based Progression Risk Stratification and Tumor Grading of Non-Muscle-Invasive Bladder Cancer
Source: Cancers (Basel). 2022 Sep 21;14(19):4570. doi: 10.3390/cancers14194570 (PMC9558547; doi:10.3390/cancers14194570)

Supplementary Figure S1

Time-to-progression curves for HER2 immunohistochemistry in the total cohort of non-muscle invasive bladder cancer patients: 0/1+/2+ vs. 3+.

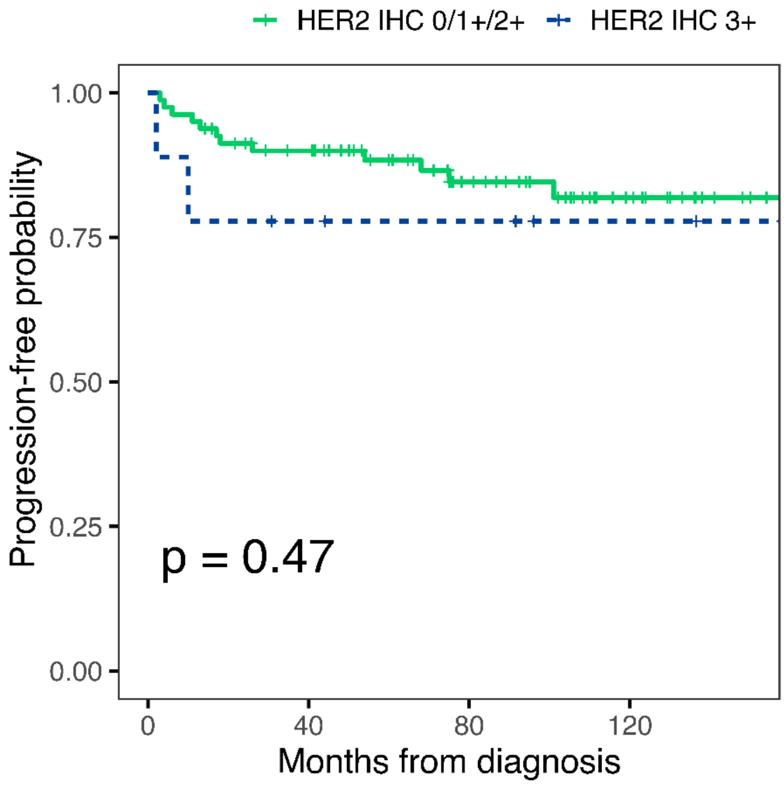

Supplement: Supplementary file 1 [file cancers-14-04570-s001.zip › Supplementary Figure S1_proof.pdf]
