# Supplementary material for: Addition of Chromosome 17 Polysomy and HER2 Amplification Status Improves the Accuracy of Clinicopathological Factor-Based Progression Risk Stratification and Tumor Grading of Non-Muscle-Invasive Bladder Cancer
Source: Cancers (Basel). 2022 Sep 21;14(19):4570. doi: 10.3390/cancers14194570 (PMC9558547; doi:10.3390/cancers14194570)

Supplementary Figure S4

Time-to-progression curves in non-muscle invasive bladder cancer patients for (A) WHO 2004/2016 low- vs. high-grade (B) Molecular low-grade vs. high-grade groups. Progressive disease is defined as progression to stage T2 or higher stage disease. P-values (log-rank test) are indicated in each figure. WHO: World Health Organization

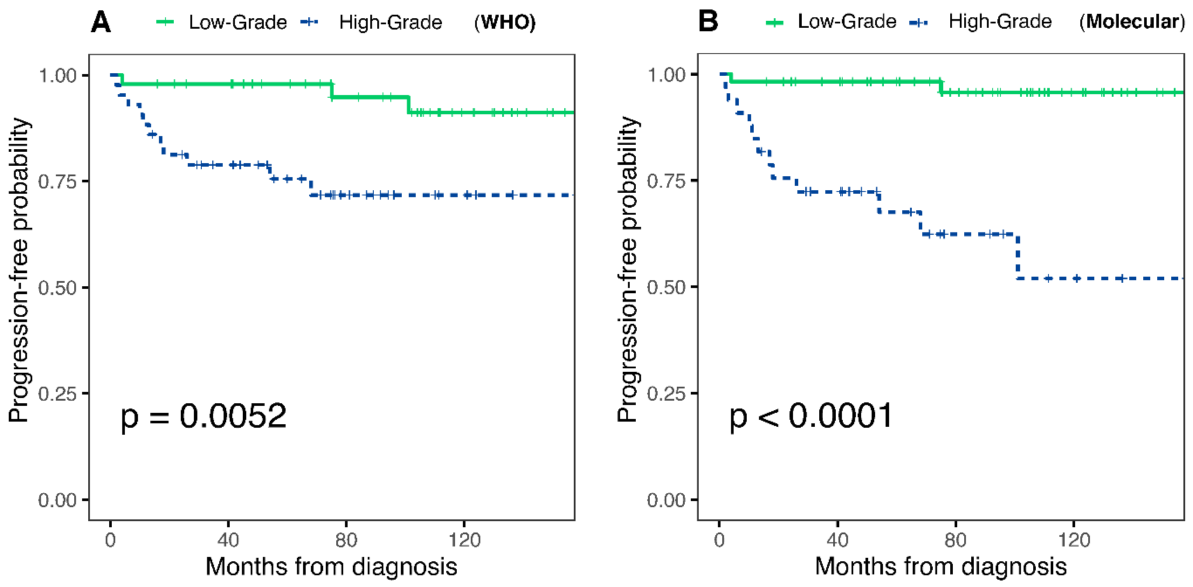

Supplement: Supplementary file 1 [file cancers-14-04570-s001.zip › Supplementary Figure S4_proof.pdf]
