# Supplementary material for: Addition of Chromosome 17 Polysomy and HER2 Amplification Status Improves the Accuracy of Clinicopathological Factor-Based Progression Risk Stratification and Tumor Grading of Non-Muscle-Invasive Bladder Cancer
Source: Cancers (Basel). 2022 Sep 21;14(19):4570. doi: 10.3390/cancers14194570 (PMC9558547; doi:10.3390/cancers14194570)
Supplement: Supplementary file 1 [file cancers-14-04570-s001.zip › Supplementary Table S1_proof.pdf]

Supplementary Table S1

Progression risk group stratification of the patients		
**EAU risk groups with the WHO 1973 classification**		
Low risk	8	(8.89)
Intermediate risk	50	(55.56)
High risk	31	(34.44)
Very high risk	1	(1.11)
**EAU risk groups with the WHO 2004/2016 classification**		
Low risk	25	(27.78)
Intermediate risk	29	(32.22)
High risk	35	(38.89)
Very high risk	1	(1.11)
**EORTC risk groups**		
Progression score=0	20	(22.22)
Progression score=2-6	43	(47.78)
Progression score=7-13	24	(26.67)
Progression score=14-23	2	(2.22)
**AUA risk groups**		
Low risk	13	(14.44)
Intermediate risk	39	(43.33)
High risk	38	(42.22)

Abbreviations: EAU, European Association of Urology; EORTC, European Organisation for Research and Treatment of Cancer; AUA, American Urological Association.
