# Supplementary material for: Addition of Chromosome 17 Polysomy and HER2 Amplification Status Improves the Accuracy of Clinicopathological Factor-Based Progression Risk Stratification and Tumor Grading of Non-Muscle-Invasive Bladder Cancer
Source: Cancers (Basel). 2022 Sep 21;14(19):4570. doi: 10.3390/cancers14194570 (PMC9558547; doi:10.3390/cancers14194570)
Supplement: Supplementary file 1 [file cancers-14-04570-s001.zip › Supplementary Table S2_proof.pdf]

Supplementary Table S2

Relation of the HER2 immunohistochemistry results and the clinicopathological characteristics of the tumors

Tumor characteristics	† HER2 IHC 0		HER2 IHC 1+		HER2 IHC 2+		HER2 IHC 3+		p	CI of OR	
	n	%	n	%	n	%	n	%		Lower	Upper
**Tumor type**											
**Primary**	21	(70.00)	14	(77.78)	27	(81.82)	8	(88.89)			
**Recurrent**	9	(30.00)	4	(22.22)	6	(18.18)	1	(11.11)	0.678	0.009	3.413
**Stage**											
**pTa**	15	(50.00)	8	(44.44)	18	(54.55)	1	(11.11)			
**pT1**	15	(50.00)	10	(55.56)	14	(42.42)	8	(88.89)	0.032*	1.027	381.370
**pTis**	0	(0.00)	0	(0.00)	1	(3.03)	0	(00.0)			
**Grade**											
**1**	10	(33.33)	4	(22.22)	6	(18.18)	0	(00.0)			
**2**	16	(53.33)	12	(66.67)	22	(66.67)	2	(22.22)	<0.001#	3.447	231.954
**3**	4	(13.33)	2	(11.11)	5	(15.15)	7	(77.78)			
**Low grade**	19	(63.33)	14	(77.78)	14	(42.42)	0	(00.0)			
**High grade**	11	(36.67)	4	(22.22)	19	(57.58)	9	(100.00)	<0.001	2.485	inf
**Tumor size**											
**<3 cm**	25	(83.33)	14	(77.78)	23	(69.70)	6	(66.67)			
**≥3 cm**	5	(16.67)	4	(22.22)	10	(30.30)	3	(33.33)	0.683	0.240	8.506
**Multiplicity**											
**Solitary**	27	(90.00)	15	(83.33)	28	(84.85)	9	(100.00)			
**Multiple**	3	(10.00)	3	(16.67)	5	(15.15)	0	(0.00)	0.594	0.000	3.782
**Recurrence**											
**Yes**	16	(53.33)	14	(77.78)	22	(66.67)	2	(22.22)			
**No**	14	(46.67)	4	(22.22)	11	(33.33)	7	(77.78)	0.027	1.077	64.502
**Progression to T2**											
**Yes**	5	(16.67)	1	(5.56)	6	(18.18)	2	(22.22)			
**No**	25	(83.33)	17	(94.44)	27	(81.82)	7	(77.78)	0.626	0.099	6.734

Tumor characteristics	Heterogeneous for HER2 overexpression		Non-heterogeneous for HER2 overexpression		p	CI of OR	
	n	%	n	%		Lower	Upper
**Tumor type**							
**Primary**	35	(85.37)	35	(71.43)			
**Recurrent**	6	(14.63)	14	(28.57)	0.133	0.730	8.223
**Stage**							
**pTa**	18	(43.90)	24	(48.98)			
**pT1**	22	(53.66)	25	(51.02)	0.831*	0.339	2.136
**pTis**	1	(2.44)	0	(0.00)			
**Grade**							
**1**	6	(14.63)	14	(28.57)			
**2**	26	(63.41)	26	(53.06)	0.793#	0.249	2.579
**3**	9	(21.95)	9	(18.37)			
**Low grade**	14	(34.15)	33	(67.35)			
**High grade**	27	(65.85)	16	(32.65)	0.003	0.095	0.659

**Tumor size**							
**<3 cm**	28	(68.29)	40	(81.63)			
**≥3 cm**	13	(31.71)	9	(18.37)	0.218	0.160	1.431
**Multiplicity**							
**Solitary**	36	(87.80)	43	(87.76)			
**Multiple**	5	(12.20)	6	(12.24)	1	0.234	4.531
**Recurrence**							
**Yes**	23	(56.10)	31	(63.27)			
**No**	18	(43.90)	18	(36.73)	0.523	0.292	1.885
**Progression to T2**							
**Yes**	5	(12.20)	9	(18.37)			
**No**	36	(87.80)	40	(81.63)	0.562	0.149	2.293

IHC: immunohistochemistry; † HER2 IHC 0/1+/2+ vs. HER2 IHC 3+; *Ta vs. T1 tumors; # Grade 1/2 vs. Grade 3
