# Supplementary material for: Addition of Chromosome 17 Polysomy and HER2 Amplification Status Improves the Accuracy of Clinicopathological Factor-Based Progression Risk Stratification and Tumor Grading of Non-Muscle-Invasive Bladder Cancer
Source: Cancers (Basel). 2022 Sep 21;14(19):4570. doi: 10.3390/cancers14194570 (PMC9558547; doi:10.3390/cancers14194570)
Supplement: Supplementary file 1 [file cancers-14-04570-s001.zip › Supplementary Table S3_proof.pdf]

Supplementary Table S3

Multivariable Cox regression analysis of potential predictor variables and time-to-progression

Variable	Category	HR	95% CI	P
<MODEL 1>				0.070
Age, years	continuous variable	1.035	(0.981-1.093)	0.203
Histologic grade (WHO 1973)	Grade 3 vs. grade 1-2 (Ref.)	2.620	(0.686-10.012)	0.159
HER2 amplified by FISH	Amplified vs. non-amplified (Ref.)	2.045	(0.399-10.474)	0.391
<MODEL 2>				**0.002***
Age, years	continuous variable	1.040	(0.981-1.103)	0.193
Histologic grade (WHO 1973)	Grade 3 vs. grade 1-2 (Ref.)	1.467	(0.455-4.731)	0.522
Chromosome 17 polysomy	≥2.25 vs. <2.25 signal/cell (Ref.)	6.433	(1.776-23.307)	**0.005***
<MODEL 3>				**0.008***
Age, years	continuous variable	1.029	(0.971-1.091)	0.337
Histologic grade (WHO 1973)	Grade 3 vs. grade 1-2 (Ref.)	1.926	(0.551-6.737)	0.305
Chromosome 17 high polysomy	≥3.45 vs. <3.45 signal/cell (Ref.)	5.012	(1.363-18.432)	**0.015***
<MODEL 4>				**0.002***
Age, years	continuous variable	1.048	(0.990-1.110)	0.106
Histologic grade (WHO 1973)	Grade 3 vs. grade 1-2 (Ref.)	1.884	(0.620-5.726)	0.264
Distinct highly polysomic cell population	Yes vs. No (Ref.)	6.179	(1.598-23.894)	**0.008***

HR: hazard ratio; CI: confidence interval
