# Supplementary material for: Addition of Chromosome 17 Polysomy and HER2 Amplification Status Improves the Accuracy of Clinicopathological Factor-Based Progression Risk Stratification and Tumor Grading of Non-Muscle-Invasive Bladder Cancer
Source: Cancers (Basel). 2022 Sep 21;14(19):4570. doi: 10.3390/cancers14194570 (PMC9558547; doi:10.3390/cancers14194570)
Supplement: Supplementary file 1 [file cancers-14-04570-s001.zip › Supplementary Table S4_proof.pdf]

Supplementary Table S4

Univariate Cox regression analysis of the progression risk scores and multivariable analysis with the potential predictor variables and time-to-progression

Variable	Category	HR	95% CI	P	C –index / corr. C-index
EAU risk groups with the WHO 1973 classification	High and very high risk vs. low and intermediate risk (Ref.)	3.814	(1.272-11.430)	**0.017***	0.684 / 0.684
<MODEL 1 EAU 1973>				**0.030***	0.699 / 0.669
EAU risk groups with the WHO 1973 classification	High and very high risk vs. low and intermediate risk (Ref.)	3.167	(0.963-10.416)	0.058	
HER2 gene amplification	Amplified vs. non-amplified (Ref.)	2.046	(0.511-8.192)	0.312	
<MODEL 2 EAU 1973>				**<0.001***	0.784 / 0.767
EAU risk groups with the WHO 1973 classification	High and very high risk vs. low and intermediate risk (Ref.)	1.989	(0.610-6.485)	0.254	
Chromosome 17 polysomy	≥2.25 vs. <2.25 signal/cell (Ref.)	5.639	(1.600-19.868)	**0.007***	
<MODEL 3 EAU 1973>				**0.003***	0.725 / 0.704
EAU risk groups with the WHO 1973 classification	High and very high risk vs. low and intermediate risk (Ref.)	2.333	(0.673-8.085)	0.182	
Chromosome 17 high polysomy	≥3.45 vs. <3.45 signal/cell (Ref.)	4.722	(1.350-16.525)	**0.015***	
<MODEL 4 EAU 1973>				**<0.001***	0.771 / 0.758
EAU risk groups with the WHO 1973 classification	High and very high risk vs. low and intermediate risk (Ref.)	2.777	(0.911-8.465)	0.073	
Distinct highly polysomic cell population	Yes vs. No (Ref.)	5.364	(1.466-19.627)	**0.011***	
EAU risk groups with the WHO 2004/2016 classification	High and very high risk vs. low and intermediate risk (Ref.)	7.086	(1.955-25.680)	**0.003***	0.741 / 0.739
<MODEL 1 EAU 2004/2016>				**0.003***	0.755 / 0.735
EAU risk groups with the WHO 2004/2016 classification	High and very high risk vs. low and intermediate risk (Ref.)	6.302	(1.654-24.016)	**0.007***	
HER2 gene amplification	Amplified vs. non-amplified (Ref.)	1.679	(0.445-6.338)	0.445	
<MODEL 2 EAU 2004/2016>				**<0.001***	0.793 / 0.788
EAU risk groups with the WHO 2004/2016 classification	High and very high risk vs. low and intermediate risk (Ref.)	3.800	(0.937-15.410)	0.062	
Chromosome 17 polysomy	≥2.25 vs. <2.25 signal/cell (Ref.)	4.197	(1.176-14.970)	**0.027***	
<MODEL 3 EAU 2004/2016>				**<0.001***	0.789 / 0.781
EAU risk groups with the WHO 2004/2016 classification	High and very high risk vs. low and intermediate risk (Ref.)	4.925	(1.256-19.310)	**0.022***	
Chromosome 17 high polysomy	≥3.45 vs. <3.45 signal/cell (Ref.)	3.688	(1.138-11.950)	**0.030***	
<MODEL 4 EAU 2004/2016>				**<0.001***	0.795 / 0.789
EAU risk groups with the WHO 2004/2016 classification	High and very high risk vs. low and intermediate risk (Ref.)	4.691	(1.244-17.690)	**0.022***	
Distinct highly polysomic cell population	Yes vs. No (Ref.)	4.261	(1.143-15.890)	**0.031***	

EORTC risk groups	Progression score <7 vs. ≥7	2.254	(0.756-6.717)	0.145	0.612 / 0.586
<MODEL 1 EORTC>				0.100	0.629 / 0.574
EORTC risk groups	Progression score <7 vs. ≥7	1.468	(0.379-5.680)	0.579	
HER2 gene amplification	Amplified vs. non-amplified (Ref.)	3.331	(0.671-16.540)	0.141	
<MODEL 2 EORTC>				**0.004***	0.763 / 0.732
EORTC risk groups	Progression score <7 vs. ≥7	1.102	(0.341-3.563)	0.871	
Chromosome 17 polysomy	≥2.25 vs. <2.25 signal/cell (Ref.)	6.670	(1.864-23.870)	**0.004***	
<MODEL 3 EORTC>				**0.030***	0.643 / 0.602
EORTC risk groups	Progression score <7 vs. ≥7	1.197	(0.314-4.559)	0.792	
Chromosome 17 high polysomy	≥3.45 vs. <3.45 signal/cell (Ref.)	5.940	(1.385-25.475)	**0.017***	
<MODEL 4 EORTC>				**0.007***	0.741 / 0.713
EORTC risk groups	Progression score <7 vs. ≥7	1.590	(0.523-4.832)	0.413	
Distinct highly polysomic cell population	Yes vs. No (Ref.)	5.579	(1.500-20.742)	**0.010***	
AUA risk groups	High vs. low and intermediate risk (Ref.)	6.252	(1.729-22.610)	**0.005***	0.728 / 0.728
<MODEL 1 AUA>				**0.005***	0.745 / 0.724
AUA risk groups	High vs. low and intermediate risk (Ref.)	5.473	(1.440-20.806)	**0.013***	
HER2 gene amplification	Amplified vs. non-amplified (Ref.)	1.844	(0.489-6.961)	0.366	
<MODEL 2 AUA>				**<0.001***	0.786 / 0.781
AUA risk groups	High vs. low and intermediate risk (Ref.)	3.123	(0.751-12.980)	0.117	
Chromosome 17 polysomy	≥2.25 vs. <2.25 signal/cell (Ref.)	4.369	(1.195-15.980)	**0.026***	
<MODEL 3 AUA>				**<0.001***	0.781 / 0.774
AUA risk groups	High vs. low and intermediate risk (Ref.)	4.322	(1.108-16.860)	**0.035***	
Chromosome 17 high polysomy	≥3.45 vs. <3.45 signal/cell (Ref.)	4.022	(1.245-12.990)	**0.020***	
<MODEL 4 AUA>				**<0.001***	0.773 / 0.767
AUA risk groups	High vs. low and intermediate risk (Ref.)	3.712	(0.961-14.350)	0.057	
Distinct highly polysomic cell population	Yes vs. No (Ref.)	4.080	(1.061-15.700)	**0.041***	
Risk groups of original EAU risk stratification	High and very high risk vs. low and intermediate risk (Ref.)	3.636	(1.013-13.060)	**0.048***	0.666 / 0.662

HR: hazard ratio; CI: confidence interval; corr. C-index: bootstrap-corrected C-index; EAU: European Association of Urology; EORTC: European Organisation for Research and Treatment of Cancer; AUA: American Urological Association. * Statistically significant p values are displayed in bold.
