# Supplementary material for: Addition of Chromosome 17 Polysomy and HER2 Amplification Status Improves the Accuracy of Clinicopathological Factor-Based Progression Risk Stratification and Tumor Grading of Non-Muscle-Invasive Bladder Cancer
Source: Cancers (Basel). 2022 Sep 21;14(19):4570. doi: 10.3390/cancers14194570 (PMC9558547; doi:10.3390/cancers14194570)
Supplement: Supplementary file 1 [file cancers-14-04570-s001.zip › Supplementary Table S5_proof.pdf]

Supplementary Table S5

Relation of the p53 immunohistochemistry results and the Chromosome 17 status of the tumors

	P53 IHC positive		P53 IHC negative		p	CI of OR	
**Chr17 status**	n	%	n	%		Lower	Upper
**Chromosome 17 polysomy**							
**Polysomic (≥ 2.25 signal/cell)**	15	(71.43)	10	(15.63)			
**Non-polysomic (<2.25 signal/cell)**	6	(28.57)	54	(84.38)	>0.001	3.708	51.618
**Chromosome 17 high polysomy**							
**Polysomic (≥ 3.45 signal/cell)**	6	(28.57)	2	(3.13)			
**Non-polysomic (<3.45 signal/cell)**	15	(71.43)	62	(96.88)	0.002	1.895	132.114
**Highly polysomic cell population**							
**Yes**	15	(71.43)	17	(26.56)			
**No**	6	(28.57)	47	(73.44)	>0.001	2.065	24.869

P53 status of a case was considered negative if the positive nuclear staining rate was between 1-49% of the total number of tumor cells (corresponding to wild type p53), while 0% and 50-100% staining rates were considered as positive p53 status (abnormal p53 IHC pattern).
