# Supplementary material for: Addition of Chromosome 17 Polysomy and HER2 Amplification Status Improves the Accuracy of Clinicopathological Factor-Based Progression Risk Stratification and Tumor Grading of Non-Muscle-Invasive Bladder Cancer
Source: Cancers (Basel). 2022 Sep 21;14(19):4570. doi: 10.3390/cancers14194570 (PMC9558547; doi:10.3390/cancers14194570)
Supplement: Supplementary file 1 [file cancers-14-04570-s001.zip › Supplementary Table S6_proof.pdf]

Supplementary Table S6

Relation of the p53 immunohistochemistry results and the other variables

p53 expression vs. other variables	non-progressive NMIBCs			Subtotal	progressive NMIBCs			Subtotal	Total
p53 positivity in % of nuclei	<1%	1-49%	50-100%		<1%	1-49%	50-100%		
pTa	3	31	4	38	-	3	-	3	41
pT1	3	25	6	34	1	5	3	9	43
pTis	-	-	-	0	-	-	1	1	1
G1	-	18	1	19	-	1	-	1	20
G2	3	33	6	42	1	5	1	7	49
G3	3	5	3	11	-	2	3	5	16
Low grade	2	38	1	41	-	3	-	3	44
High grade	4	18	9	31	1	5	4	10	41
EAU 2004 low risk (1-2)	3	40	5	48	-	3	-	3	51
EAU 2004 high risk (3-4)	3	16	5	24	1	5	4	10	34
Polysomy 17	3	6	7	16	1	4	4	9	25
High polysomy 17	1	1	2	4	1	1	3	5	9
Highly polysomic cell population	2	12	8	22	1	5	4	10	32
HER2 amplification	1	1	2	4	-	2	1	3	7
Polysomy 17 and/or *HER2* amplification	3	6	7	16	1	4	4	9	25
Non-amplified, non-polysomic	3	50	3	56	-	4	-	4	60
HER2 IHC score 0-1+	4	34	2	40	1	4	-	5	45
HER2 IHC score 2+	1	20	5	26	-	3	3	6	32
HER2 IHC score 3+	1	2	3	6	-	1	1	2	8
